# Small-Field Measurements of 3D Polymer Gel Dosimeters through Optical Computed Tomography

**DOI:** 10.1371/journal.pone.0151300

**Published:** 2016-03-14

**Authors:** Tian-Yu Shih, Jay Wu, Cheng-Ting Shih, Yao-Ting Lee, Shin-Hua Wu, Chun-Hsu Yao, Bor-Tsung Hsieh

**Affiliations:** 1 Graduate Institute of Clinical Medical Science, China Medical University, Taichung, Taiwan; 2 Department of Medical Imaging and Radiological Science, Central Taiwan University of Sciences and Technology, Taichung, Taiwan; 3 Department of Radiology, Cheng Ching Hospital at Chung Kang, Taichung, Taiwan; 4 Department of Biomedical Imaging and Radiological Sciences, National Yang-Ming University, Taipei, Taiwan; 5 3D Printing Medical Research Center, China Medical University Hospital, Taichung, Taiwan; 6 Department of Biomedical Imaging and Radiological Science, College of Health Care, China Medical University, Taichung, Taiwan; 7 Department of Biomedical Informatics, Asia University, Taichung, Taiwan; Chongqing University, CHINA

## Abstract

With advances in therapeutic instruments and techniques, three-dimensional dose delivery has been widely used in radiotherapy. The verification of dose distribution in a small field becomes critical because of the obvious dose gradient within the field. The study investigates the dose distributions of various field sizes by using NIPAM polymer gel dosimeter. The dosimeter consists of 5% gelatin, 5% monomers, 3% cross linkers, and 5 mM THPC. After irradiation, a 24 to 96 hour delay was applied, and the gel dosimeters were read by a cone beam optical computed tomography (optical CT) scanner. The dose distributions measured by the NIPAM gel dosimeter were compared to the outputs of the treatment planning system using gamma evaluation. For the criteria of 3%/3 mm, the pass rates for 5 × 5, 3 × 3, 2 × 2, 1 × 1, and 0.5 × 0.5 cm^2^ were as high as 91.7%, 90.7%, 88.2%, 74.8%, and 37.3%, respectively. For the criteria of 5%/5 mm, the gamma pass rates of the 5 × 5, 3 × 3, and 2 × 2 cm^2^ fields were over 99%. The NIPAM gel dosimeter provides high chemical stability. With cone-beam optical CT readouts, the NIPAM polymer gel dosimeter has potential for clinical dose verification of small-field irradiation.

## Introduction

At present, there are three major ways to treat malignant tumors: surgical operation, radiotherapy, and chemotherapy. Modern radiotherapy techniques can maximize the radiation dose to tumors and minimize the one applied to normal tissue by using small-field segments of a multileaf collimator. In addition, radiometric tools play a crucial role in accurately verifying the radiation dose and irradiation scope. Gel dosimeters provide three-dimensional (3D) dose information, which can be used as a validation of treatment planning for patients.

The low-toxicity *N*-isopropylacrylamide (NIPAM) polymer gel dosimeter proposed by Senden et al. [1] has been intensively researched for the optimization of its formula [2] and its readout systems. The results showed that the NIPAM gel dosimeter had a high linear dose response ranging from 2–15 Gy [3], and can be read out by various scanning tools [4,5]. During gel reading, the changes of proton groups in gels can be measured using magnetic resonance imaging (MRI), and the T2 results can be correlated with the 3D dose distribution [6,7]. In recent years, several researchers measured the degree of polymerization in gels using computed tomography (CT). The absorbed dose is proportional to the CT number of gels [8,9]. Gore et al. [10] designed an optical computed tomography (optical CT) to evaluate the fundamental characteristics of polymer gel dosimeters. The absorbed dose can be quantified by measuring the difference in luminous intensity of laser beams through the gel.

Dose evaluation of small-field irradiation techniques, such as radiosurgery and stereotactic radiotherapy, is an important issue to provide a better treatment quality. Currently, only a few studies focused on gel dosimetric measurements of small-field dose delivery. Olding et al. [11] used the NIPAM-based gel dosimeter to measure two letter dose patterns and read the gel by a cone beam optical CT. The results of gamma evaluation with 2%/2 mm criteria showed that the dose maps had a 92.7% pass rate compared to the result of the treatment planning system (TPS). Hassani et al. [12] measured 6-MV X-ray beams with field sizes of 5 × 5, 10 × 10, 20 × 20, and 30 × 30 mm^2^ using the methacrylic and ascorbic acid in gelatin initiated by copper (MAGIC) polymer gel dosimeter, and the dose distribution was read out by MRI. The dose profiles of the MAGIC gel dosimeter had a maximum difference of 2.08% compared to those of the verification films at the edge of the penumbra. These studies only investigate 1D profiles or 2D planes. There is still a lack of overall 3D evaluation on small-field dose delivery by polymer gel dosimeters.

In this study, the NIPAM polymer gel dosimeters were used to measure the dose distributions of various small field sizes. The gel dosimeters were read out by using a commercial cone beam optical CT, and the dose distributions were compared to the results of TPS using gamma evaluation.

## Materials and Methods

### Preparation of the Polymeric Gel Dosimeter

NIPAM based gel dosimeters were fabricated using the gelatin (300 Bloom Type A, Sigma-Aldrich, St Louis, MO), NIPAM as the monomer (97% Wako, Osako, Japan), *N*,*N*’-methylene-bis-acrylamide (BIS) as the crosslinking agent (Sigma-Aldrich, St Louis, MO), and antioxidant tetrakis (hydroxymethyl) phosphonium chloride (THPC) as the antioxidant agent (TCI, Sigma-Aldrich, St Louis, MO). The weight percentages are listed in Table 1 [13]. The gelatin was added to deionized water, and the solution was stirred for 10 min at room temperature. The solution was then heated to 45°C. After it became transparent, the NIPAM and BIS were added and stirred until the components were dissolved. Then, the THPC was added and stirred for another 2 min. At last, the polymer gels were poured into a cylindrical container with a diameter and height of 10 cm. The NIPAM gel dosimeters were kept at 4°C for 6 hours before irradiation [14,15].

**Table 1:** Formula of the NIPAM polymer gel dosimeter.

Composition	Weight (%)	Amount^a^
**Gelatin**	5	5 ± 0.0001 g
**Monomer: NIPAM**	5	5 ± 0.0001 g
**Cross linker: BIS**	3	3 ± 0.0001 g
**Distilled water**	87	87 ± 0.1 mL
**THPC (mM)**	5	8.96×10^−2^ ± 1×10^−3^ mL^b^

^a^ The amount of the composition is measured for preparing a 100-mL NIPAM dosimeter.

^b^ THPC is measured using micropipettes.

### Irradiation

**Fig 1:**
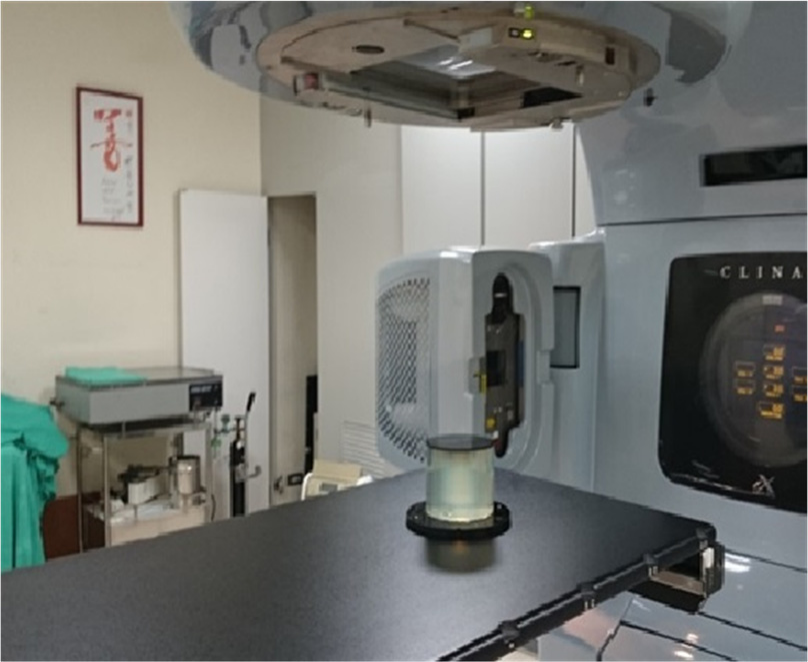
Dose delivery to the NIPAM gel dosimeter. The irradiation parameters were 6-MV photo beams, 400 MU/min, 98.5-cm source to surface distance (SSD), and 0° gantry angle.

The dose delivery planning was performed using the Eclipse TPS (Varian Medical Systems, Palo Alto, CA) with 1-mm-thick CT images of the gel dosimeters. A single small field was irradiated using a Varian Clinac iX linear accelerator (Varian Medical Systems, Palo Alto, CA) for a prescribed dose of 5 Gy at the center of the gel container. The irradiation parameters were 6-MV photo beams, 400 MU/min, 98.5-cm source to surface distance (SSD), and 0° gantry angle (Fig 1). Five filed sizes were evaluated, including 5 × 5, 3 × 3, 2 × 2, 1 × 1, and 0.5 × 0.5 cm^2^. Five batches of the NIPAM gel dosimeters were fabricated; the variation between each batch was lower than 1% [14].

### Gel Dosimeter Reading

In this study, a cone beam optical CT scanner (VISTA, Modus Medical Devices Inc., USA) was used for the gel dosimeter readout. In this method, a light-emitting diode matrix was used as a 633-nm light source. The laser beam penetrates through the water bath, matching fluid and gel, and then is collected by a charge coupled device (CCD) camera. After beam data acquisition, the cross-sectional images of the gels can be reconstructed. The spatial resolution of the reconstructed optical CT images is 0.81 mm/pixel. In this study, the NIAPM polymer gel dosimeters were scanned 24, 48, 72, and 96 hours after dose delivery. The light attenuation coefficient was calculated by the following equation:

$$\alpha =-\frac{1}{\chi }\text{ln}\left[\frac{I}{I_{0}}\right]$$ (1)

where *χ* is the diameter of the gel container. *Ι*_0_ and *I* are the intensities of the incident and penetrated laser beams, respectively. The dose conversion and data analysis were performed using MATLAB (The Math Works, Natick, MA).

## Results and Discussion

**Fig 2:**
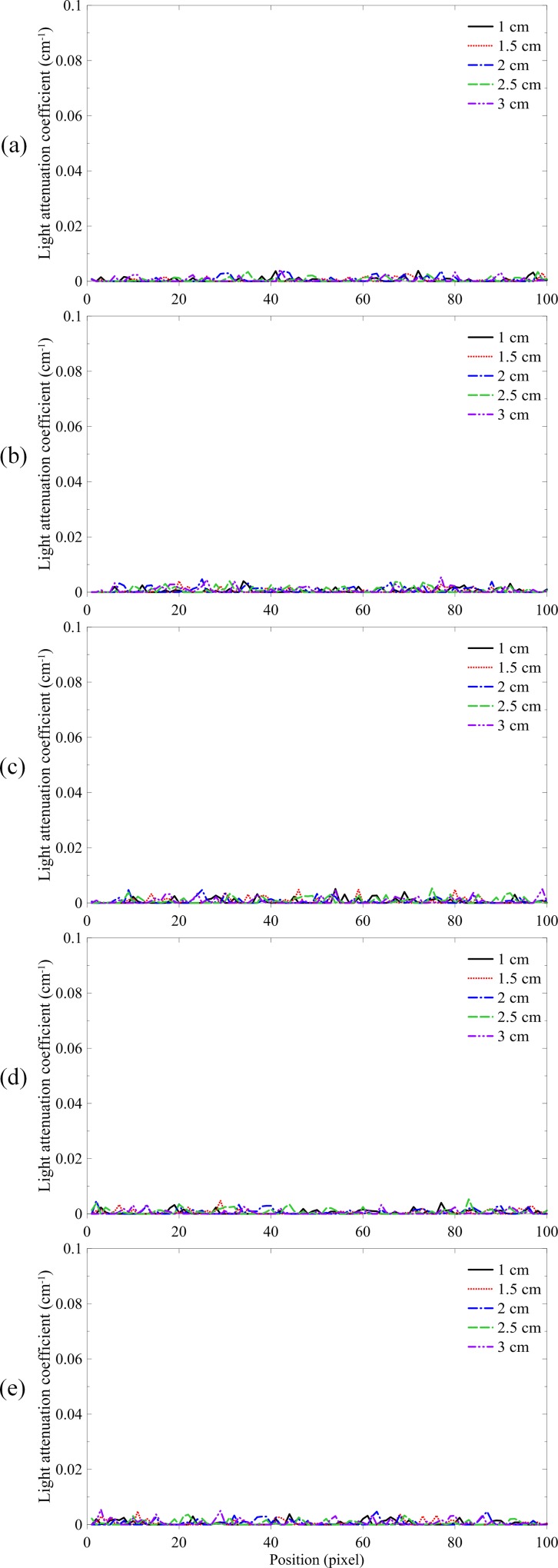
Light attenuation coefficient profiles of the non-irradiated NIPAM gel dosimeters at depths from 10 mm to 30 mm. (a) Batch 1, (b) batch 2, (c) batch 3, (d) batch 4, and (e) batch 5 were analyzed.

The non-irradiated NIPAM gel dosimeters were scanned six hours after fabrication. Five depths were acquired, including 10, 15, 20, 25, and 30 mm. Fig 2 shows the light attenuation coefficient profiles of five batches of the non-irradiated NIPAM gel dosimeters. The uniformity of the five batches was 0.17%, 0.20%, 0.17%, 0.19%, and 0.18%, respectively, indicating a high chemical stability of the NIPAM gel dosimeter.

**Fig 3:**
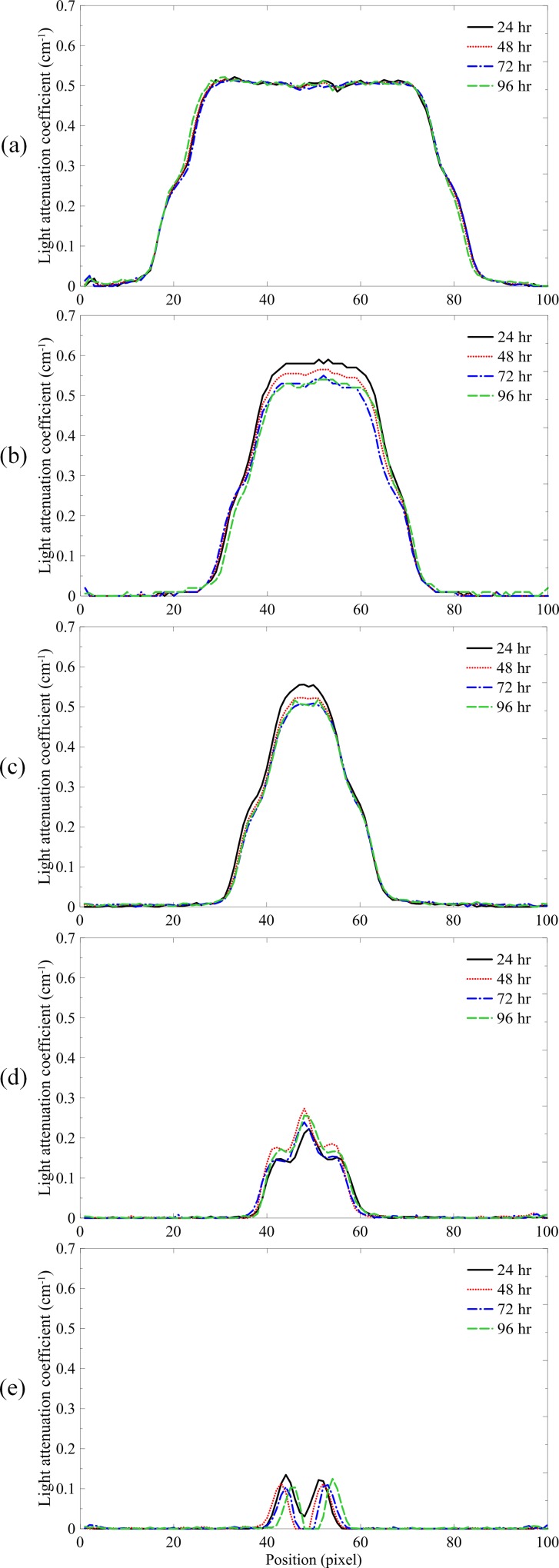
Light attenuation coefficient profiles of the NIPAM gel dosimeters. The gels were irradiated by (a) 5 × 5, (b) 3 × 3, (c) 2 × 2, (d) 1 × 1, and (e) 0.5 × 0.5 cm^2^ field sizes.

Fig 3 shows the profiles of the 24-, 48-, 72-, and 96-hour images of the NIPAM gel dosimeter irradiated with field sizes of 5 × 5, 3 × 3, 2 × 2, 1 × 1, and 0.5 × 0.5 cm^2^. The average percent standard deviations in the central area for the five field sizes were 1.61%, 1.30%, 1.59%, 15.8%, and 140.0%, respectively. The polymerization process of the NIPAM gel dosimeter required at least 24 to 48 hours to stabilize the reaction. This result matches with the previous experiment [15]. Moreover, the stable condition maintained at least for 96 hours, indicating that the NIPAM gel dosimeter has a mild diffusion effect after the polymerization is completed. In other words, the NIPAM gel dosimeter has high stability and repeatability.

**Fig 4:**
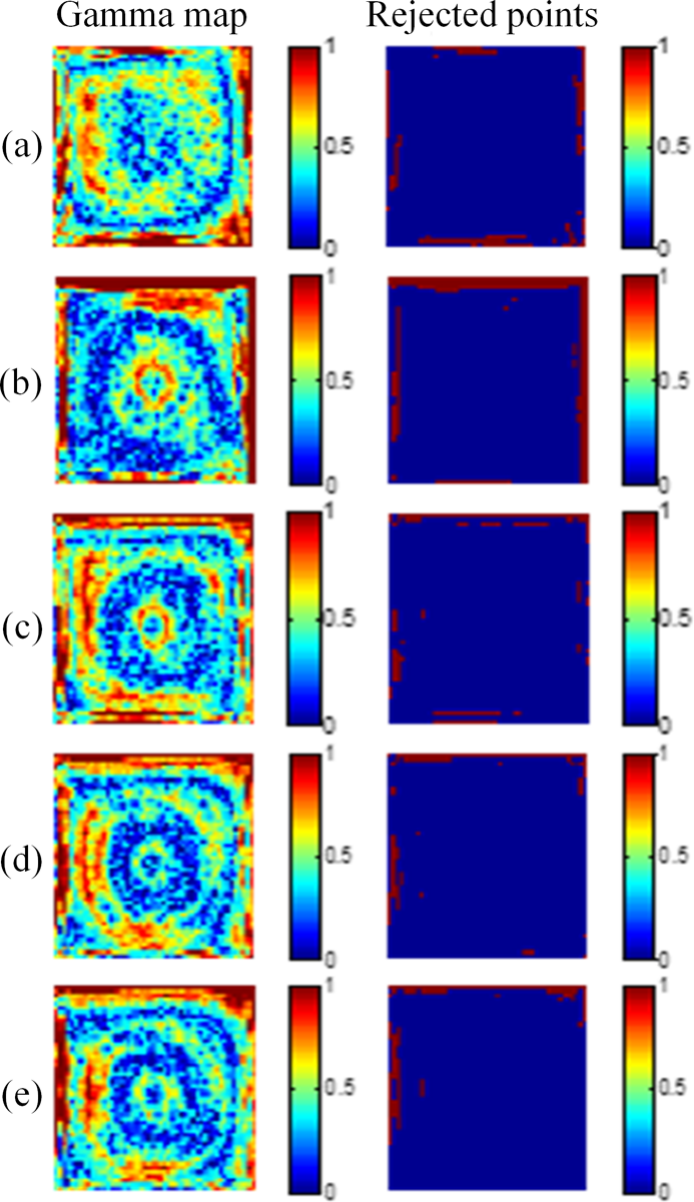
Gamma maps of the 5 × 5 cm^2^ field size. The criteria of 3%/3 mm were applied at depths of (a) 10 mm, (b) 15 mm, (c) 20 mm, (d) 25 mm, and (e) 30 mm.

**Fig 5:**
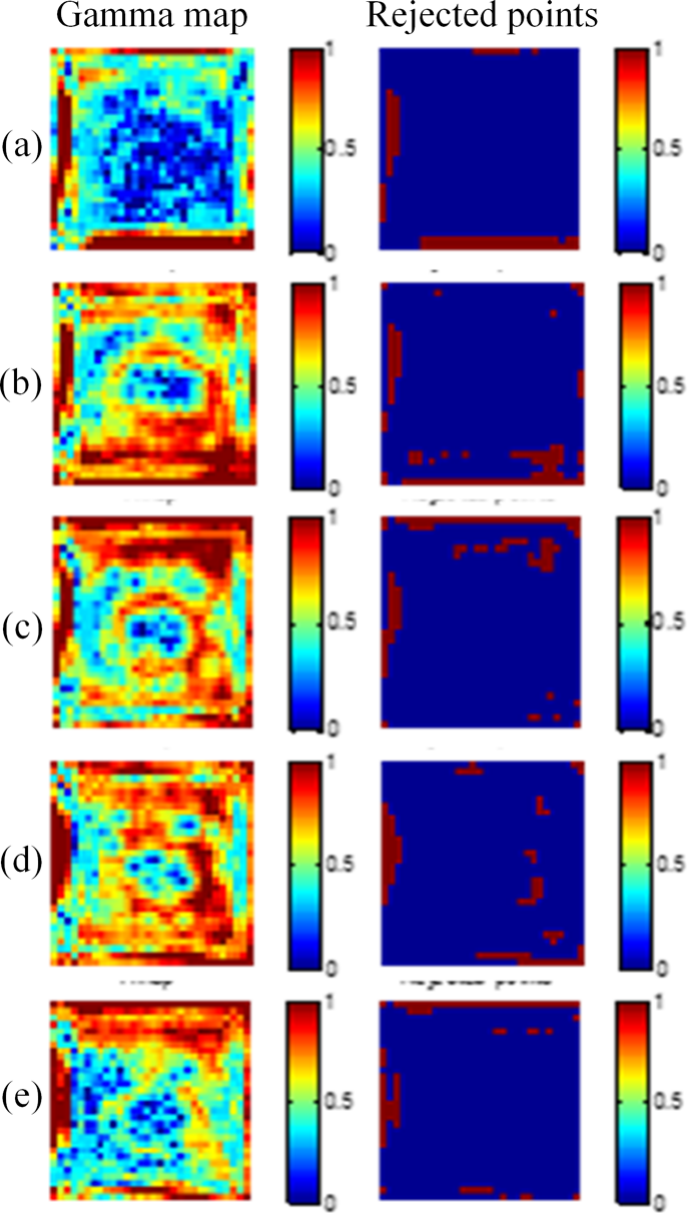
Gamma maps of the 3 × 3 cm^2^ field size. The criteria of 3%/3 mm were applied at depths of (a) 10 mm, (b) 15 mm, (c) 20 mm, (d) 25 mm, and (e) 30 mm.

By using the gamma evaluation, we compared the dose maps converted from the optical CT images with the results of TPS. The criteria of the gamma evaluation were 3%/3 mm for the region within the 50% isodose line. Fig 4 displays the gamma maps of 10-mm, 15-mm, 20-mm, 25-mm, and 30-mm depths with the 5 × 5 cm^2^ field size. The pass rates of the five depths were 91.3%, 91.4%, 91.7%, 91.4%, and 91.1%, respectively. Fig 5 shows the results for the 3 × 3 cm^2^ field size, and the pass rates of the five depths were 90.6%, 89.7%, 89.9%, 90.4%, and 90.7%, respectively.

The comparison of dose distributions between the gel dosimeter measurements and the TPS results showed that the points failing to pass the criteria (index > 1) were mainly occurred at the edge of the field, where the dose gradient was steep. This is mainly caused by the edge enhancement characteristic of the NIPAM gel dosimeter. Polymers around the irradiated region diffuse inward and are polymerized [15]. Another factor may come from the slight mismatch of the spatial resolution between the optical CT image and the TPS. The coarse grid of the TPS jeopardizes the gamma pass rate.

**Fig 6:**
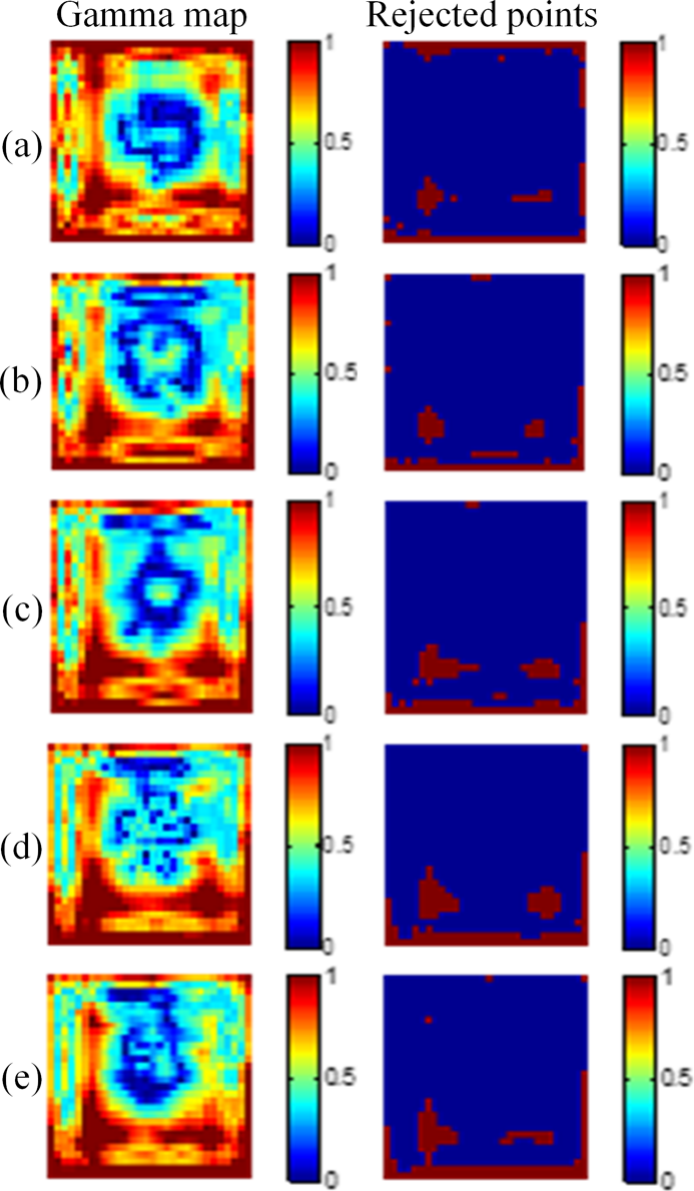
Gamma maps of the 2 × 2 cm^2^ field size. The criteria of 3%/3 mm were applied at depths of (a) 10 mm, (b) 15 mm, (c) 20 mm, (d) 25 mm, and (e) 30 mm.

**Fig 7:**
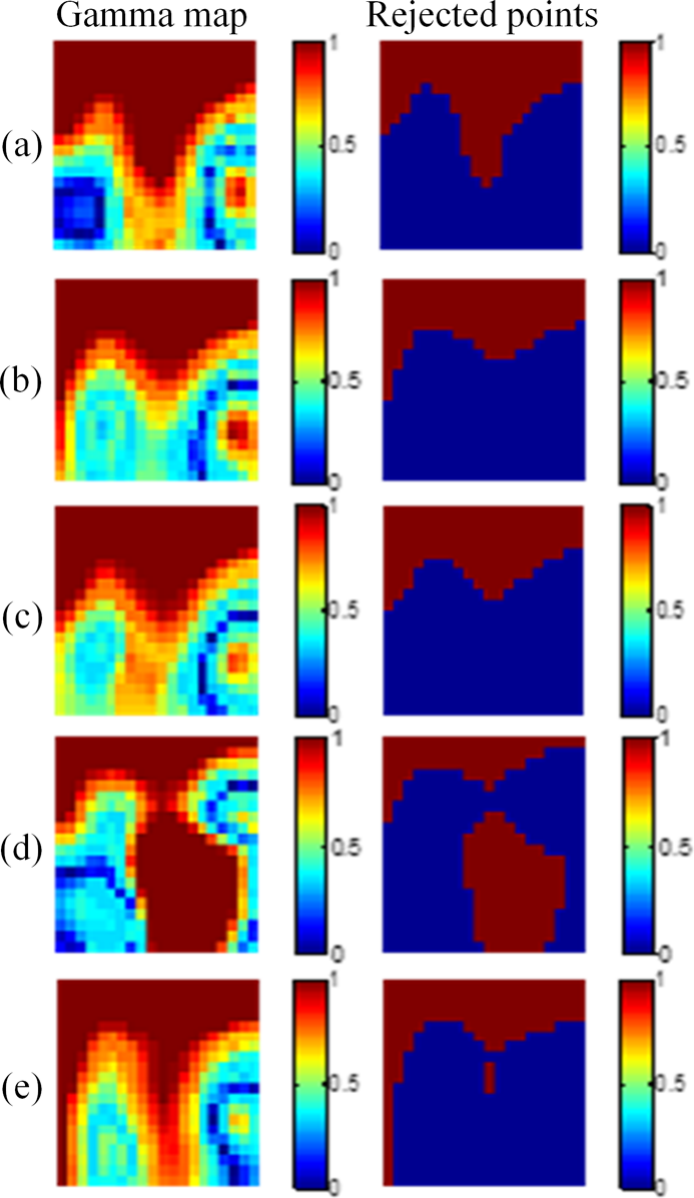
Gamma maps of the 1 × 1 cm^2^ field size. The criteria of 3%/3 mm were applied at depths of (a) 10 mm, (b) 15 mm, (c) 20 mm, (d) 25 mm, and (e) 30 mm.

**Fig 8:**
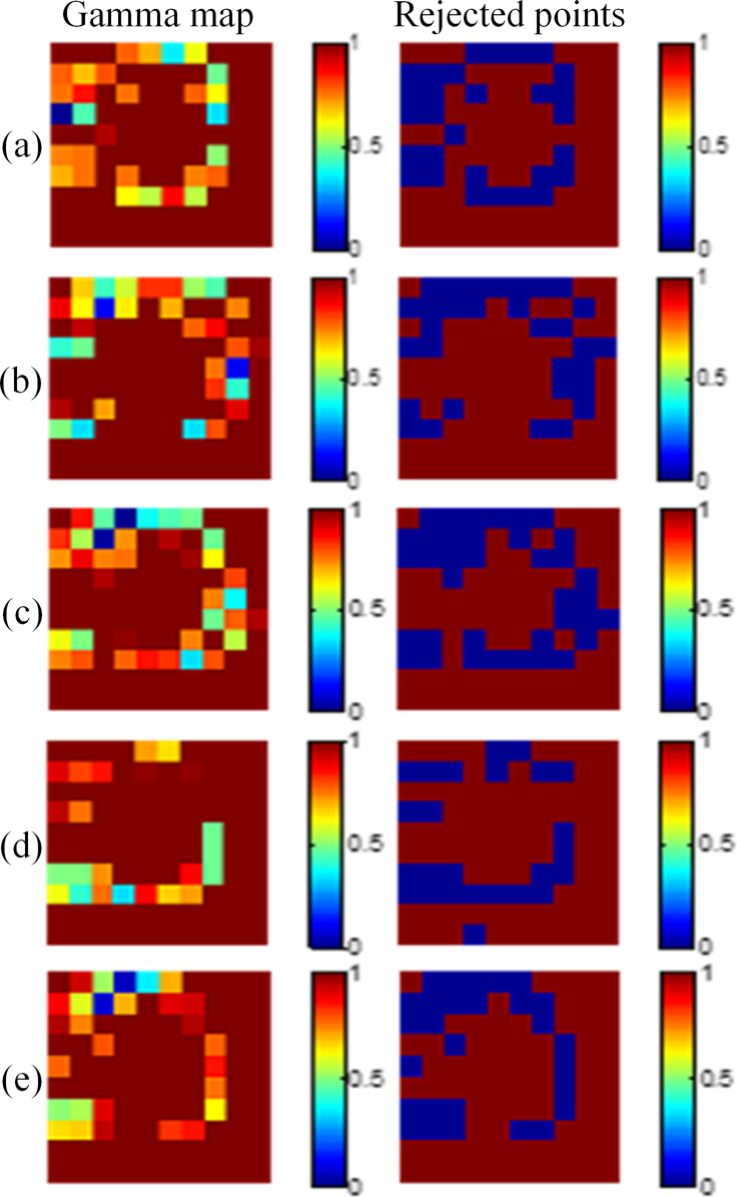
Gamma maps of the 0.5 × 0.5 cm^2^ field size. The criteria of 3%/3 mm were applied at depths of (a) 10 mm, (b) 15 mm, (c) 20 mm, (d) 25 mm, and (e) 30 mm.

Fig 6 shows the results of the 2 × 2 cm^2^ field size. The pass rates of the five depths were 87.2%, 88.1%, 88.2%, 86.2%, and 86.1%, respectively. Fig 7 shows the results of the 1 × 1 cm^2^ field. The pass rates were 74.5%, 74.8%, 73.6%, 74.2%, and 73.4%, respectively. Fig 8 shows the results of the 0.5 × 0.5 cm^2^ field. The pass rates dramatically decreased to 29.0%, 31.1%, 37.3%, 25.5%, and 28.4%, respectively.

Tables 2–6 list the pass rates of various field sizes and gamma criteria measured 24, 48, 72, and 96 hours after irradiation. When the field size was larger than 3 × 3 cm^2^, the pass rates with criteria of 3%/3 mm, 4%/4 mm, and 5%/5 mm were 88%–92%, 94%–98%, and above 99%, respectively. The pass rates of the 2 × 2 cm^2^ field with criteria of 3%/3 mm, 4%/4 mm, and 5%/5 mm were 83%–88%, 95%–97%, above 99%, respectively. The gamma pass rates of the 3 × 3 cm^2^ field was generally 2% to 3% higher than those of the 2 × 2 cm^2^ field due to the lower perimeter/area ratio of the irradiated region. Additionally, the differences of pass rates for various scan times were generally within 2%. These results indicate the stability of the irradiated gel dosimeters with no further polymerization.

**Table 2:** Pass rates of the 5 × 5 cm^2^ field size measured 24–96 hours after irradiation at depths of 10–30 mm.

Time (hr)	10 mm	15 mm	20 mm	25 mm	30 mm
3%/3 mm					
24	91.3%	91.4%	91.7%	91.4%	91.1%
48	91.6%	90.8%	90.0%	90.3%	90.3%
72	91.7%	91.4%	91.2%	89.2%	90.0%
96	90.6%	90.5%	90.9%	90.8%	90.5%
4%/4 mm					
24	97.3%	95.1%	98.3%	98.5%	98.3%
48	98.2%	95.5%	95.3%	96.6%	96.8%
72	98.3%	95.4%	95.7%	97.1%	97.4%
96	94.7%	94.8%	96.6%	97.0%	97.5%
5%/5 mm					
24	99.2%	99.8%	100.0%	99.9%	99.9%
48	99.4%	99.5%	99.5%	99.8%	99.6%
72	99.5%	99.4%	99.6%	99.1%	99.4%
96	99.8%	99.8%	99.5%	99.2%	99.8%

**Table 3:** Pass rates of the 3 × 3 cm^2^ field size measured 24–96 hours after irradiation at depths of 10–30 mm.

Time (hr)	10 mm	15 mm	20 mm	25 mm	30 mm
3%/3 mm					
24	90.6%	89.7%	89.9%	90.4%	90.7%
48	89.8%	90.0%	88.1%	89.3%	89.0%
72	91.3%	91.3%	91.4%	91.0%	92.1%
96	91.7%	90.2%	90.0%	91.1%	91.2%
4%/4 mm					
24	97.4%	98.9%	98.6%	98.3%	98.7%
48	97.7%	98.4%	98.4%	98.7%	98.6%
72	98.3%	98.3%	98.0%	98.8%	98.6%
96	98.0%	98.6%	98.2%	98.1%	98.4%
5%/5 mm					
24	99.0%	99.7%	99.9%	99.9%	99.9%
48	99.4%	99.9%	99.8%	99.7%	99.5%
72	99.4%	99.5%	99.3%	99.1%	99.4%
96	99.4%	99.4%	99.4%	99.7%	99.6%

**Table 4:** Pass rates of the 2 × 2 cm^2^ field size measured 24–96 hours after irradiation at depths of 10–30 mm.

Time (hr)	10 mm	15 mm	20 mm	25 mm	30 mm
3%/3 mm					
24	87.2%	88.1%	88.2%	86.2%	86.1%
48	87.9%	88.6%	86.5%	89.1%	89.8%
72	87.2%	88.3%	88.3%	87.4%	87.6%
96	83.7%	84.9%	85.3%	84.2%	85.3%
4%/4 mm					
24	97.0%	97.5%	96.1%	97.5%	97.3%
48	97.6%	97.5%	97.0%	97.4%	97.7%
72	97.0%	96.7%	97.4%	96.6%	97.2%
96	95.1%	96.1%	96.0%	95.8%	96.0%
5%/5 mm					
24	99.3%	99.1%	99.3%	99.5%	99.1%
48	99.6%	99.5%	99.8%	99.8%	99.5%
72	99.5%	99.6%	99.8%	99.7%	99.9%
96	99.8%	99.5%	99.2%	99.1%	99.3%

**Table 5:** Pass rates of the 1 × 1 cm^2^ field size measured 24–96 hours after irradiation at depths of 10–30 mm.

Time (hr)	10 mm	15 mm	20 mm	25 mm	30 mm
3%/3 mm					
24	74.5%	74.8%	73.6%	74.2%	73.4%
48	74.9%	72.7%	76.3%	74.5%	74.7%
72	74.4%	77.9%	73.3%	77.2%	76.4%
96	73.0%	74.8%	73.6%	76.3%	75.7%
4%/4 mm					
24	85.3%	84.8%	85.3%	85.6%	86.1%
48	85.4%	82.8%	85.8%	89.5%	85.4%
72	87.3%	87.2%	86.2%	87.4%	87.2%
96	87.1%	84.8%	84.9%	88.0%	86.8%
5%/5 mm					
24	93.1%	91.4%	92.6%	92.4%	93.4%
48	93.0%	90.1%	93.4%	94.1%	93.3%
72	94.3%	93.6%	93.2%	93.5%	94.4%
96	94.2%	93.3%	93.2%	94.7%	95.0%

**Table 6:** Pass rates of the 0.5 × 0.5 cm^2^ field size measured 24–96 hours after irradiation at depths of 10–30 mm.

Time (hr)	10 mm	15 mm	20 mm	25 mm	30 mm
3%/3 mm					
24	29.0%	31.1%	37.3%	25.5%	28.4%
48	31.6%	38.1%	38.2%	27.8%	33.7%
72	26.4%	34.3%	38.7%	32.9%	34.9%
96	31.5%	32.1%	36.3%	31.0%	32.7%
4%/4 mm					
24	44.3%	56.8%	57.1%	48.8%	52.0%
48	54.8%	59.3%	56.4%	57.2%	55.3%
72	49.9%	54.6%	55.4%	52.8%	57.1%
96	56.4%	53.3%	62.3%	54.0%	53.5%
5%/5 mm					
24	64.5%	66.8%	69.3%	72.1%	72.2%
48	75.1%	71.3%	77.1%	72.6%	76.2%
72	65.7%	73.2%	72.6%	71.7%	72.1%
96	72.4%	68.8%	80.2%	78.7%	72.5%

The pass rates of the 1 × 1 cm^2^ field with 3%/3 mm, 4%/4 mm, and 5%/5 mm criteria were 73%–77%, 82%–87%, and 90%–95%, respectively. The pass rates of 0.5 × 0.5 cm^2^ field with 3%/3 mm, 4%/4 mm, and 5%/5 mm criteria were 25%–38%, 44%–62%, and 64%–80%, respectively. The points that failed to pass the gamma criteria now propagated from the marginal region of the field to the center of the field. The variations between scan times increased substantially.

In this study, the gamma pass rates of small fields are not satisfactory. The possible reasons are the chemical characteristics of polymer gel dosimeters. According to the previous study [3], the NIPAM based gel dosimeters have a linear dose response of 2−15 Gy and a minor dose rate dependence of approximately 30%. In the penumbra region of small fields, the dose distribution has a steep slope because of the low scattering compared to one in the central axis. Since the prescribed dose in the center of the field was 5 Gy, the dose and dose rate near the edge may be lower than the applicable range of the NIPAM gel dosimeters. This phenomenon can also be observed in the percent depth dose (PDD) measurement using the NIPAM gel cassette [16], where the PDD of the gel dosimeter was significantly lower than the result of the ion chamber at the build-up region. Fortunately, the dose information in the penumbra region has a low effect on the treatment quality. If we constrain the gamma evaluation region within 90% of the isodose line, the gamma pass rate could be largely improved. However, if the dose distribution in the buildup region or penumbra is interested, a more sensitive gel dosimeter, such as the methacrylic acid-based gel (nMAG) [17], should be used.

Errors may also come from several physical factors. The TPS utilizes the beam data measured by a farmer-type ion chamber as its basis for dose calculations. The ion chamber has its own uncertainty, and the errors inevitably propagate to the dose results through the calculation algorithm. Additionally, the scatter perturbation during optical CT scans is an inherent problem of optical CT, even though refractive index matching fluid is added. Findings by other researchers also suffered from these discrepancies [12].

## Conclusion

This study verifies the small-field dose distributions of radiotherapy using NIPAM gel dosimeters with cone beam optical CT readouts. The findings showed that the gamma pass rates for the field size larger than 3 × 3 cm^2^ were 88–92%, 94–98%, and 99% for criteria of 3%/3 mm, 4%/4 mm, and 5%/5 mm, respectively. The pass rates for the field size smaller than 3 × 3 cm^2^ were generally lower than 90% for criteria of 3%/3 mm, which reaches an unacceptable level for clinical radiotherapy. As a conclusion, the NIPAM polymer gel dosimeter provides high chemical stability. With cone-beam optical CT readouts, the NIPAM gel dosimeter has great potential for clinical dose verification. The unsatisfactory pass rates on the field size smaller than 3 cm pose a challenge to conquer in the future.
